# The novel exchange protein activated by cyclic AMP 1 (EPAC1) agonist, I942, regulates inflammatory gene expression in human umbilical vascular endothelial cells (HUVECs)

**DOI:** 10.1016/j.bbamcr.2018.11.004

**Published:** 2019-02

**Authors:** Jolanta Wiejak, Boy van Basten, Urszula Luchowska-Stańska, Graham Hamilton, Stephen J. Yarwood

**Affiliations:** a Institute of Biological Chemistry, Biophysics and Bioengineering, School of Engineering and Physical Sciences, Heriot-Watt University, Edinburgh Campus, Edinburgh EH14 4AS, UK; b Glasgow Polyomics, Wolfson Wohl Cancer Research Centre, Garscube Campus, University of Glasgow, Bearsden G61 1QH, UK

**Keywords:** Vascular endothelial cells, Inflammation, Transcriptome, Cyclic AMP, EPAC1, Cell adhesion molecules

## Abstract

Exchange protein activated by cyclic AMP (EPAC1) suppresses multiple inflammatory actions in vascular endothelial cells (VECs), partly due to its ability to induce expression of the suppressor of cytokine signalling 3 (SOCS3) gene, the protein product of which inhibits interleukin 6 (IL6) signalling through the JAK/STAT3 pathway. Here, for the first time, we use the non-cyclic nucleotide EPAC1 agonist, I942, to determine its actions on cellular EPAC1 activity and cyclic AMP-regulated gene expression in VECs. We demonstrate that I942 promotes EPAC1 and Rap1 activation in HEK293T cells and induces SOCS3 expression and suppresses IL6-stimulated JAK/STAT3 signalling in HUVECs. SOCS3 induction by I942 in HUVECs was blocked by the EPAC1 antagonist, ESI-09, and EPAC1 siRNA, but not by the broad-spectrum protein kinase A (PKA) inhibitor, H89, indicating that I942 regulates SOCS3 gene expression through EPAC1. RNA sequencing was carried out to further identify I942-regulated genes in HUVECs. This identified 425 I942-regulated genes that were also regulated by the EPAC1-selective cyclic AMP analogue, 007, and the cyclic AMP-elevating agents, forskolin and rolipram (F/R). The majority of genes identified were suppressed by I942, 007 and F/R treatment and many were involved in the control of key vascular functions, including the gene for the cell adhesion molecule, VCAM1. I942 and 007 also inhibited IL6-induced expression of VCAM1 at the protein level and blocked VCAM1-dependent monocyte adhesion to HUVECs. Overall, I942 represents the first non-cyclic nucleotide EPAC1 agonist in cells with the ability to suppress IL6 signalling and inflammatory gene expression in VECs.

## Highlights

• The novel EPAC1 ligand I942 activates cellular EPAC1 and Rap1 GTPase.
• I942 induces SOCS3 gene expression in vascular endothelial cells (VECs).
• I942 suppresses JAK/STAT3 signalling from the IL6 receptor in VECs.
• I942 regulates 425 novel gene targets in VECs.
• I942 suppresses VCAM1 expression and monocyte adhesion in VECs.

## Graphical abstract

**Figure:**
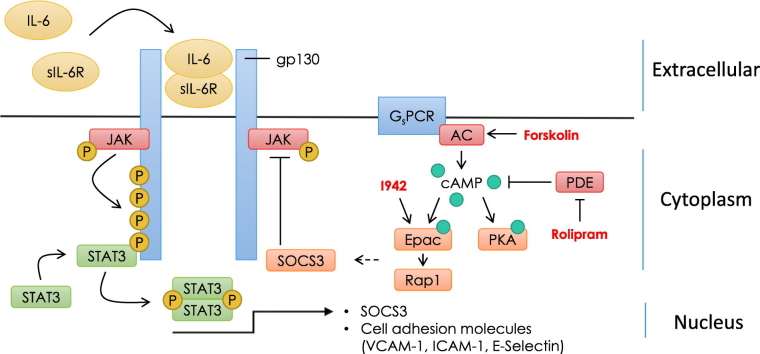

## 1 Introduction

Atherosclerosis is a serious cardiovascular disease (CVD), which arises from chronic localised inflammation at coronary and carotid arterial branch points [1], and remains the principal cause of death in the developed world (http://www.who.int/cardiovascular_diseases/en/) despite changes in lifestyle and the widespread use of anti-hypertensive and lipid-lowering drugs. The increased inflammatory activity associated with atherosclerosis is partially brought about by increased levels of pro-inflammatory cytokines in the circulation, particularly IL6 [2,3], which is associated with a twofold increase in CVDs and mortality in elderly patients [4]. IL6 localises to atherosclerotic plaques [5] and promotes chronic, low-level vascular inflammation, leading to neointimal thickening [6], vascular dysfunction [7], hypertension [8] and increased risk of myocardial infarction [2]. IL6 affects vascular endothelial cells (VECs) by triggering counter-productive angiogenesis, through vascular endothelial growth factor (VEGF) production [9], and increasing the secretion of chemokines, like the chemokine (C-C motif) ligand 2 (CCL2), also referred to as monocyte chemo-attractive protein 1 (MCP-1) [10], that recruit monocytes to the inflamed endothelium. Initial engagement of monocytes with the endothelium involves rolling adhesion mediated by E-selectin (SELE) [11,12], followed by firm adhesion mediated by interactions between integrins expressed on leukocytes and adhesion molecules (ICAM1, VCAM1) expressed on endothelial cells [12]. Finally, cadherin molecules at adherens junctions mediate trans-endothelial migration of monocytes [13,14].

It is IL6 receptor “trans-signalling” [15] that is thought to underlie the pro-inflammatory actions of IL6 in a variety of diseases, including atherosclerosis [16]. During trans-signalling, IL6 binds to soluble forms of the IL6 receptor α (sIL6Rα), allowing activation of gp130, even in cells that do not normally express the IL6Rα subunit, such as VECs [15]. Since gp130 is present on all cells, trans-signalling therefore dramatically increases the range of cell targets for IL6 signalling to include VECs [15]. Consequently, binding of the IL6/sIL6R complex to gp130 on VECs leads to receptor clustering and activation of the JAK/STAT3 and ERK, MAPK and PI3K signalling pathways. Of these, it is activated STAT3 that then homodimerises and translocates to the nucleus, where it acts as a transcription factor for the induction of pro-inflammatory IL6-responsive genes, such as CCL2/MCP1, VEGF, VCAM1, ICAM1 and SELE [10,[17], [18], [19]]. IL6-promoted JAK/STAT3 signalling is normally downregulated by the suppressor of cytokine signalling (SOCS) family of proteins [20], which are induced by JAK/STAT3, forming a classical negative feedback loop [21]. The SOCS3 protein contains an SH2 domain that interacts with JAK-phosphorylated receptors, thereby inhibiting JAK/STAT3 signalling [22]. SOCS3 expression is increased in atherosclerotic plaques [23,24] and the importance of SOCS3 for the suppression of CVD has been shown by a series of knockdown experiments, which demonstrate that IL6 promotes acute and chronic inflammatory disease in the absence of SOCS3 [25]. Moreover, knockdown of SOCS3 in VECs leads to pathological angiogenesis [26] and enhanced STAT3 activation and atherogenesis in apolipoprotein-E (apoE)^−/−^ mice [24]. In contrast, overexpression of SOCS3 peptide fragments or full-length protein suppresses JAK/STAT3 signalling and the development of atherosclerosis [[27], [28], [29], [30]]. SOCS3 induction may therefore serve as a valid drug target for the suppression of inflammation associated with atherogenesis.

The cyclic AMP sensor enzyme, EPAC1, is a logical drug target for vascular inflammation because its ligand-mediated activation suppresses a wide range of inflammatory actions [[31], [32], [33]]. EPAC1 and EPAC2 proteins [34,35] are cyclic AMP-regulated guanine nucleotide exchange factors (GEFs) that activate the Ras GTPase homologues, Rap1 and Rap2, independently of the classical route of cAMP signal transduction through protein kinase A (PKA). Rather, binding of cAMP to a specific cyclic nucleotide-binding domain (CNBD) induces a conformational change in EPAC proteins that relieves auto-inhibition of the catalytic GEF domain [33]. EPAC1 appears to be a central controller of anti-inflammatory processes in VECs [31,36]. For example, activation of EPAC1 leads to SOCS3 induction, blockade of IL6 JAK/STAT3 signalling and potentiation of barrier function in vitro and in vivo, in normal mice [36]. Moreover, activation of EPAC in cardiac myocytes attenuates the inhibitory effect of IL6 on cardiac function and contractility in response to isoproterenol, most likely through inhibition of the JAK/STAT3 signalling by SOCS3 [37]. Using high throughput screening (HTS) we identified a novel ligand (I942) that exhibits agonist properties against EPAC1, but not EPAC2. I942 interacts with the EPAC1 CNBD and activates EPAC1 GEF activity [33,38]. In contrast, there was very little agonist action of I942 towards EPAC2 or protein kinase A (PKA) [38]. This is the first identified non-cyclic nucleotide small molecule with agonist properties towards EPAC1, which can be useful as an experimental tool to investigate the role of EPAC1 in health and disease. Our aim now is to obtain evidence that I942 acts as an agonist towards EPAC1 in cells and influences inflammatory signalling in vascular endothelial cells.

## 2 Materials and methods

### 2.1 Materials

Pooled human umbilical vascular endothelial cells (HUVECs) and endothelial cell growth medium 2 (EGM2) were purchased from PromoCell (Heidelberg, Germany). Antibodies to EPAC1 (5D3), Rap1, phospho-CREB (Ser 133), STAT3/phospho-STAT3 (Tyr 705), ICAM1, VCAM1 and GAPDH were obtained from New England Biolabs UK Ltd. (Hertfordshire, UK). Antibodies to E-selectin (SELE) were from Bio-Techne (Abingdon, UK). Anti-SOCS-3 antibodies were purchased from Santa Cruz Biotechnology (Santa Cruz, CA). SuperSignal™ West Pico Chemiluminescent Substrate was from Fisher Scientific (Loughborough, UK). Secondary antibodies, anti-rabbit-IgG horseradish peroxidase, anti-goat-IgG horseradish peroxidase and anti-mouse-IgG horseradish peroxidase conjugates, were from Sigma-Aldrich Company Ltd. (Dorset, England). Forskolin, rolipram and *N*-benzoyloxycarbonyl (Z)-Leu-Leu-leucinal (MG132) were obtained from Calbiochem (Paisley, UK), whereas H-89 was from Sigma-Aldrich, UK. The EPAC1-selective cyclic AMP analogue, 8-pCPT-2′-O-Me-cAMP (007), and the EPAC1 antagonist, ESI-09, were purchased from Biolog Life Sciences Institute (Bremen, Germany). I942 (*N*-(2,4-dimethylbenzenesulfonyl)-2-(naphthalen-2-yloxy)acetamide) was purchased from MolPort (Riga, Latvia). Adrenomedullin 2/intermedin (ADM2) was from the Peptide Institute (Osaka, Japan). Recombinant human interleukin 6 (IL6) protein and recombinant human soluble IL6 receptor α (sIL6Rα) proteins were purchased from R and D Systems (Abingdon, UK).

### 2.2 Cell culture

Stably transfected Human Embryonic Kidney (HEK293T) cells, expressing 3xFlag-myc-CMV-26 vector (Sigma-Aldrich, UK) containing full-length human EPAC1, as described [39,40], were grown in Dulbecco's modified Eagle's medium (DMEM), 10% (v/v) foetal bovine serum (Sigma-Aldrich, UK), 1% (v/v) of GlutaMAX supplement (Sigma-Aldrich, UK) and 1% (v/v) penicillin/streptomycin (Sigma-Aldrich, UK) and incubated at 37 °C in 5% (v/v) CO_2_. Selection of stable cell lines was maintained by addition of 1 mg/ml G418 (Sigma-Aldrich, UK) to the growth medium. The human monocytic cell line THP-1 was cultured in RPMI 1640 medium (Fisher Scientific, UK) containing 10% (v/v) foetal bovine serum (Sigma-Aldrich, UK), 1% (v/v) penicillin/streptomycin (Sigma-Aldrich, UK) and 2 mM glutamine at 37 °C in 5% (v/v) CO_2_. HUVECs were grown in EGM2 (PromoCell) at 37 °C and 5% (v/v) CO_2_. Cells were passaged weekly to a maximum of six passages.

### 2.3 EPAC1 siRNA procedures

HUVECs were seeded at 70,000–100,000 cells per well of a 6-well culture plate and grown in 2 ml complete growth medium until 50–60% confluent. The EPAC1 siRNA (Qiagen FlexiTube Hs_RAPGEF3_5) or non-targeting siRNA (Qiagen AllStars Negative Control) was prepared for transfection by mixing 7 μl PromoFectin-HUVEC solution (Promocell) in 100 μl serum-free culture medium with 12 μl siRNA (from 20 μM stock), also dissolved in 100 μl serum-free medium, and then incubating for 20 min at room temperature. Following this, the cell culture medium was replaced with 0.9 ml fresh serum-free medium and the siRNA/PromoFectin-HUVEC solution (200 μl) was added dropwise, while gently shaking the plate, giving a final siRNA concentration of 200 nM. Cells were then incubated at 37 °C in 5% (v/v) CO_2_ for 4 h. After incubation, the medium was removed carefully by aspiration and then 2 ml of fresh complete growth medium was added, and cells were further incubated for 48 h, after which experimental procedures were carried out.

### 2.4 Western blotting

For western blotting, cells were harvested by scraping directly into 150 μl of SDS-polyacrylamide gel electrophoresis sample buffer [62.5 mM Tris-HCl, pH 6.8, 2% (w/v) SDS, 10% (v/v) glycerol, 10 mM DTT, and 0.01% (w/v) bromophenol blue]. Samples were mixed by vortexing, denaturated for 5 min at 95 °C, separated on 10% or 12% (w/v) resolving gels and then electroblotted onto nitrocellulose membranes. Membranes were then blocked in 5% (w/v) milk powder, or 5% (w/v) bovine serum albumin for phospho-specific antibodies, in Tris-buffered saline containing 0.1% (v/v) Tween 20. Blots were incubated in primary antibodies overnight at 4 °C followed by appropriate horseradish peroxidase-conjugated secondary antibodies for 1 h at room temperature. Blots were then developed using SuperSignal™ West Pico Chemiluminescent Substrate and visualised using a Fusion FX7-SPECTRA system (Vilber, Germany) fitted with a CCD camera.

### 2.5 Immunoprecipitation of active EPAC1

HEK-293T cells transfected to overexpress EPAC1 [41] were cultured in 6-well plates in HUVEC growth medium (Promocell) or Dulbecco's modified Eagle's medium (DMEM), high glucose, supplemented with 10% (v/v) FBS, 1% (v/v) GlutaMAX, 1% (v/v) penicillin/streptomycin and 1 mg/ml G418 (to ensure selection of stable transfectants), respectively, until 90% confluence was reached. Cells were then stimulated for 30 min with either 50 μM 007 or 100 μM I942. After stimulation, cells were washed with ice cold 1× PBS and lysed in 1× RIPA buffer containing 1× protease inhibitor cocktail (Roche). The activation-selective EPAC1 mouse monoclonal antibody (5D3; 2 μl) [40,43] was then added to the lysate and further incubated for 30 min (4 °C, rotation). Following this, 10 μl of protein G magnetic beads (New England Biolabs) was added to antibody-containing lysates followed by 1 h further incubation at 4 °C with rotation. Protein G magnetic beads were then captured using a magnetic separation rack and then washed three times in 1× RIPA buffer. The beads were then resuspended in SDS-polyacrylamide gel electrophoresis sample buffer and boiled for 5 min before analysis by western blotting.

### 2.6 Rap1 activation assay

**Fig. 1:**
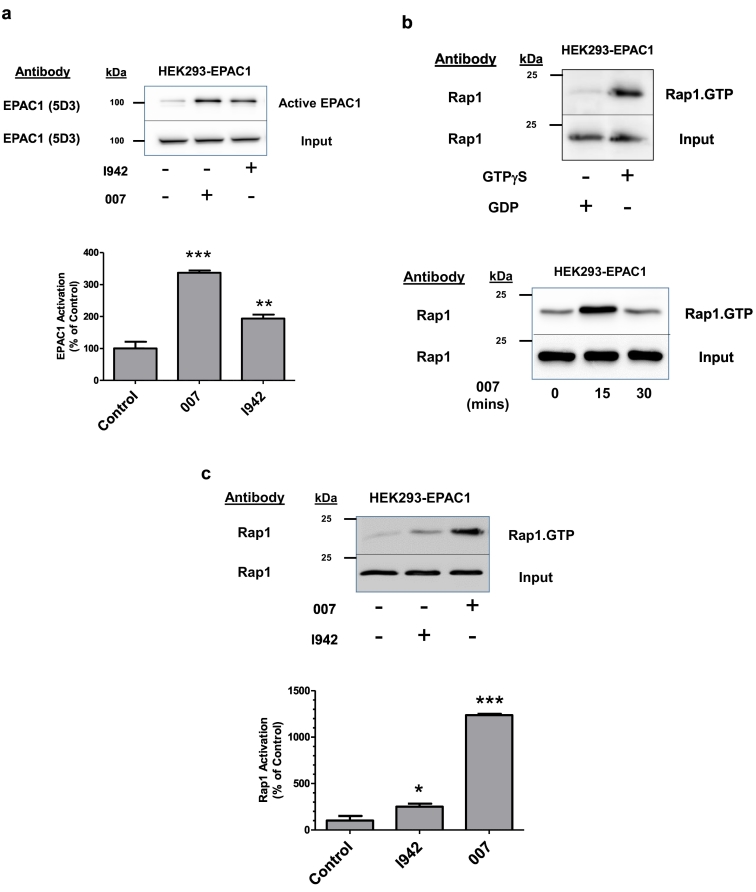
The novel EPAC1 ligand, I942, activates EPAC1 and Rap1 in HEK293T cells. a) EPAC1-expressing HEK293TT (HEK293T-EPAC1) cells were incubated with either I942 (100 μM) or 007 (50 μM) for 30 min and then cell extracts were immunoprecipitated with the anti-EPAC1 antibody, 5D3, which selectively interacts with the active form of EPAC1. The amount of immunoprecipitated, active EPAC1 in cell extracts was determined by western blotting with the 5D3 antibody (*upper panel*), with increased EPAC1 immunoreactivity indicating an increase in cellular EPAC1 activation. Multiple western blots were analysed by densitometry and the results are displayed as a histogram of means ± S.E.M. in the *lower panel*. Significant increases in EPAC1 activity, relative to diluent-treated control cells, are indicated; **, p < 0.01 and ***, p < 0.001, respectively (n = 3). b) Active Rap1 was isolated from HEK293T-EPAC1 cell extracts stimulated with either GDP or GTPγS using affinity purification columns as described in Materials and methods. The amount of purified active Rap1 in stimulated samples was determined by western blotting with an anti-Rap1 antibody (*upper panel*). In the *lower panel*, HEK293T-EPAC1 cells were stimulated for the indicated time with 007 (50 μM) and then active, Rap1.GTP, was isolated from cell extracts as described in Materials and methods. c) HEK293T-EPAC1 cells were incubated with either I942 (100 μM) or 007 (50 μM) for 30 min and then Rap1.GTP was isolated from cell extracts using affinity purification columns and visualised by immunoblotting with an anti-Rap1 antibody as described in Materials and methods. Multiple western blots were analysed by densitometry and the results are displayed as a histogram of means ± S.E.M. in the *lower panel*. Significant increases in Rap1 activity, relative to diluent-treated control cells, are indicated; *, p < 0.05 and ***, p < 0.001, respectively (n = 4).

Detection of active GTP-bound Rap1 in EPAC1-expressing HEK293TT cells was done using an Active Rap1 Detection Kit (New England Biolabs, UK) according to the manufacturer's instructions. Briefly, cells were grown to confluence in 10 cm culture dishes at 37 °C in a 5% (v/v) CO_2_ incubator. Cells were then incubated with either I942 (100 μM) or 007 (50 μM) for 15 min. Cells were then scrapped into 5 ml of 1× PBS, transferred to 15 ml centrifuge tubes and centrifuged at 800 ×*g*_max_ for 3 min at room temperature. Cell pellets were then resuspended in 0.5 ml ice-cold lysis buffer containing 1 mM PMSF, transferred to Eppendorf tubes and then lysed by vortexing. Cell lysates were kept on ice for 5 min and then centrifuged at 16000 ×*g*_max_ at 4 °C for 15 min. The supernatants were transferred to fresh Eppendorf tubes and used for GTPase assay (50 μl of each cell lysate was left for analysis of total Rap1 content). GTPγS and GDP-treated lysates were used as positive and negative controls for the assay, respectively (Fig. 1b). For the assay, cell lysates were incubated with 100 μl of 50% (w/v) glutathione-agarose beads and 20 μg of GST-RalGDS-RBD (which selectively binds Rap1.GTP) on a spin column at 4 °C for 1 h (with gentle rocking). The beads were harvested by centrifugation at 6000 ×*g*_max_ for 30 s and washed three times in 1× lysis/binding/wash buffer. GTP-bound Rap1 proteins were eluted from pelleted beads by adding 50 μl of 2× SDS Sample Buffer (containing 200 mM DTT) to the column followed by vortexing. Beads were then pelleted by centrifugation 6000 ×*g*_max_ for 2 min and eluted samples were then heated for 5 min at 95 °C and subjected to SDS-PAGE and western blot analysis with anti-Rap1 specific antibodies.

### 2.7 mRNA extraction

HUVECs were grown on 6-well plates until they had achieved 70–80% confluence. Cells were then incubated in the presence or absence of 50 μM 007 for various times up to 48 h. Total RNA was then isolated from cells using an RNeasy Kit (Qiagen, Manchester, UK), according to the manufacturer's protocol. RNA concentration was determined using NanoDrop Spectrophotometer (Thermo Fisher Scientific, Paisley, UK). Isolated total RNA was used for RNA sequencing and quantitative Real-Time PCR analysis.

### 2.8 RNA sequencing (RNAseq)

Sequencing libraries were prepared from total RNA using the Illumina TruSeq Stranded mRNA Sample Preparation Kit. Libraries were sequenced in 75 base, paired end mode on the Illumina NextSeq 500 platform. Raw sequence reads were trimmed for contaminating sequence adapters and poor quality bases using the program Cutadapt1 [44]. Bases with an average Phred score lower than 28 were trimmed. Reads that were trimmed to <54 bases were discarded. The quality of the reads was checked using the Fastqc program (http://www.bioinformatics.babraham.ac.uk/projects/fastqc/) before and after trimming. The reads were “pseudo aligned” to the transcriptome using the program Kallisto2 [45]. The differential expression for the analysis groups were assessed using the Bioconductor package DESeq23 [46]. Grouping of gene expression data into similarly responsive patterns was done using Java TreeView and CLUSTER 3.0 [47], which are freely available online (http://bonsai.hgc.jp/~mdehoon/software/cluster/software.htm).

### 2.9 Quantitative real-time PCR

For reverse-transcription, 1 μg of total RNA was converted to first-strand cDNA using RT^2^ First Strand Kit (Qiagen, UK) in accordance with the manufacturer's instructions. First, genomic DNA was eliminated using the buffer provided in the kit (5 min at 42 °C), followed by reverse-transcription reaction (15 min at 42 °C followed by 5 min at 95 °C). Real-Time PCR analysis was performed using the Qiagen Human Endothelial Cell Biology RT^2^ Profiler PCR Array (384-well format containing 4 × 96 PCR arrays) and RT^2^ SYBR Green Mastermix (Qiagen, UK) using a 7900HT Fast Real-Time PCR System (Thermo Fisher Scientific, UK), according to the manufacturer's protocol. Each PCR Array included 89 validated qPCR Primers Assays, including 5 housekeeping genes and a control panel. The thermal cycling program was as follows: 10 min at 95 °C for HotStart DNA Taq Polymerase activation, 40 cycles of denaturation at 95 °C for 15 s and annealing and extension at 60 °C for 1 min. Each experiment was run in triplicate. Data analysis and quantification of relative mRNA gene expression were performed by the ΔΔCT method using free PCR Array Data Analysis Web portal at http://pcrdataanalysis.sabiosciences.com/pcr/arrayanalysis.php.

### 2.10 Monocyte adhesion assay

Adhesion Assays were performed using Endothelial Cell Adhesion Assay Kit (Chemicon). HUVEC cells were seeded on 96-well plates at 10^4^ cells per well and incubated at 37 °C and 5% (v/v) CO_2_ overnight. The next day cells were pre-treated with I942 (100 μM) for 30 min followed by 48 h treatment with IL6/sIL6Rα. After the treatment HUVECs were incubated with blocking monoclonal antibodies against SELE, VCAM1, ICAM1 or control mouse IgG (10 μg/ml) for 1 h and then the cell adhesion assay was performed. THP-1 monocytes were labelled with the fluorescent dye calcein AM (5 μM) for 30 min, washed 3 times with PBS and resuspended in 5 ml of Assay Buffer. Cells were counted and diluted to a concentration of 2 × 10^6^ cells/ml. Calcein AM-labelled THP-1 cells were added (10^6^ cells/ml) to monolayers of HUVECs and incubated for 1 h at 37 °C in 5% (v/v) CO_2_. Non-adherent THP-1 cells were washed off with 3 times gentle washing with assay buffer. Monocyte adhesion was measured using a fluorometer with excitation and emission filters set to 494 and 517 nm, respectively.

### 2.11 Densitometry and statistical analysis

Non-saturated immunoblots from multiple experiments (n = 3) were quantified densitometrically using ImageJ software (http://rsbweb.nih.gov/ij/). Statistical significance was determined by one-way ANOVA with Tukey post-hoc test, using InStat Software (GraphPad Software, San Diego, CA).

## 3 Results

### 3.1 I942 induces cellular EPAC1 activity and Rap1 activation in HEK293T cells

We have previously shown that the EPAC1/Rap1 pathway mediates the induction of the SOCS3 gene in HUVECs, independently of PKA activation [48,49]. To confirm that the recently identified EPAC1 agonist, I942 [38], is also able to activate the EPAC1/Rap1 pathway in cells we performed EPAC1 and Rap1 activation assays in HEK293T cells stably expressing EPAC1 (HEK293T-EPAC1) [39] (Fig. 1). First, HEK293T-EPAC1 cells were stimulated with the EPAC1-selective cAMP analogue 8-pCPT-2′-O-Me-cAMP (007) [50] or I942 and then cell extracts were immunoprecipitated with the activation-selective EPAC1 monoclonal antibody, 5D3 [40,43], which has been shown to interact with active EPAC1 in these cells [40] (Fig. 1a). Both 007 and I942 treatment were found to promote a significant increase in EPAC1 activation in HEK293T-EPAC1 cells, as illustrated by an increase in the amount of immunoprecipitated EPAC1 from stimulated cells (Fig. 1a), demonstrating for the first time that I942 exerts agonist activity towards cellular EPAC1.

We next examined whether EPAC1 activation by I942 is sufficient to activate Rap1 in HEK293T-EPAC1 cells (Fig. 1b). Active, GTP-bound Rap1 (Rap1.GTP) was isolated from cell extracts by pull-down with a GST-tagged recombinant version of the Rap1-binding domain (RBD) of the protein RalGDS, which selectively interacts with Rap1.GTP [51]. Following pull-down with GSH-agarose, samples were boiled in SDS sample buffer and the amount of isolated Rap1.GTP was estimated by western blotting with an anti-Rap1 antibody (Fig. 1b). Initial experiments demonstrated that maximal activation of Rap1 occurred following 15 min stimulation with 007 (Fig. 1b, *lower panel*), achieving levels roughly equivalent to those achieved by GTPγS-stimulation of cell extracts, which represents maximal Rap1 activation by this assay (Fig. 1b, *upper panel*). Using this protocol, we observed a modest, but significant increase in Rap1 activation after 15 min treatment with 100 μM I942 (Fig. 1c), which represented around 25% of maximal Rap1 activation by 007, indicating that EPAC1 activation by I942 is able to stimulate Rap1 activity in these cells.

### 3.2 I942 promotes EPAC1-dependent SOCS3 induction and inhibition of IL6 signalling in HUVECs

**Fig. 2:**
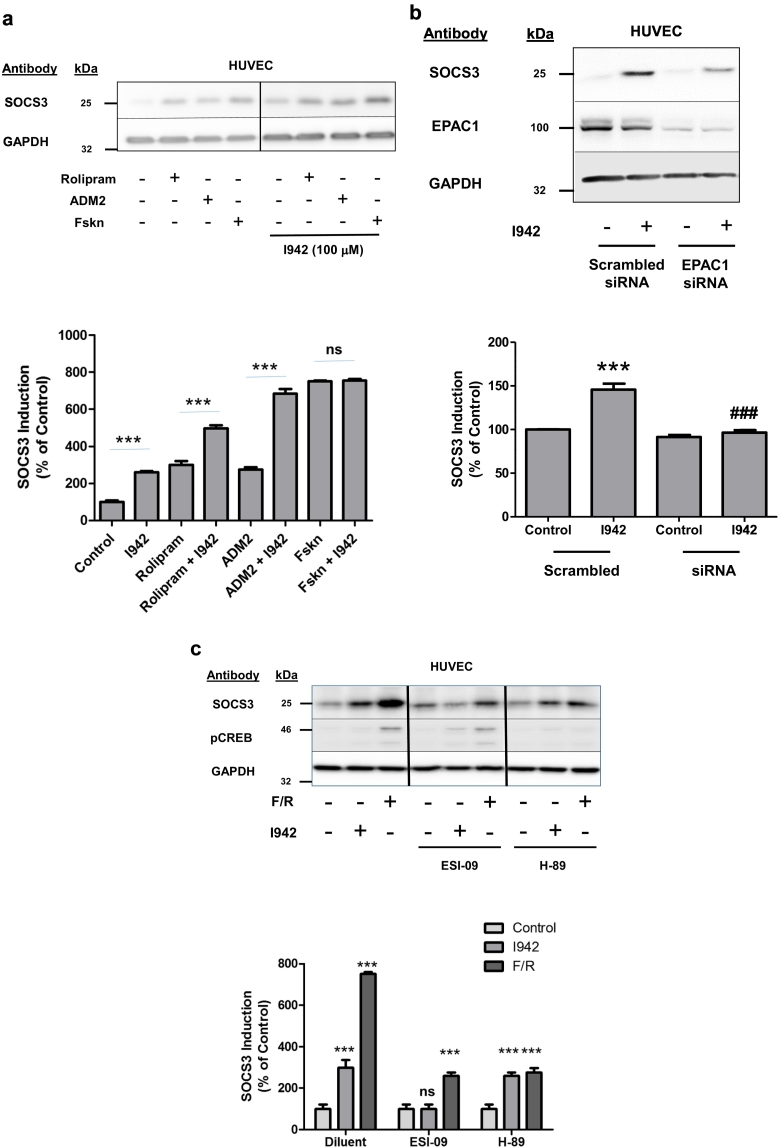
I942 activates EPAC1-dependent signalling in HUVECs. a) HUVECs were stimulated for 5 h with the indicated combinations of 10 μM forskolin, 10 μM rolipram, 100 nM ADM2 and 100 μM I942 in the presence 10 μM MG132. Cell extracts were then prepared and immunoblotted with anti-SOCS3 and anti-GAPDH antibodies. The histogram in the *lower panel* demonstrates changes in SOCS3 expression relative to control cells for three separate experiments. Significant increases in SOCS3 protein expression in I942-treated cells are indicated; ***, p < 0.001 (n = 3). Non-significant changes in SOCS3 immunoreactivity in cells treated with I942 and forskolin are also indicated (ns). b) Confluent HUVECs were pre-incubated with siRNA to EPAC1 or non-targeting, scrambled siRNA for 24 h, after which cells were treated with the proteasome inhibitor, 10 μM MG132 (to prevent breakdown of cellular SOCS3 protein), and then stimulated for 5 h in the presence or absence of 100 μM I942. Cell extracts were then prepared and immunoblotted with antibodies to SOCS3 protein, EPAC1 and GAPDH, as a loading control. Densitometry was then carried out on 3 western blots and results are shown as a histogram in the *lower panel*. Significant increases in SOCS3 protein expression, relative to diluent-stimulated control cells, are indicated; ***, p < 0.001. Significant inhibition of SOCS induction relative to I942-treated cells is also indicated; ^###^, p < 0.001. c) HUVECs were preincubated for 30 min with the EPAC1 antagonist, ESI-09 (10 μM) or the broad-spectrum protein kinase A (PKA) inhibitor, H89 (10 μM), and then stimulated for a further 5 h with either 100 μM I942 or a combination of 10 μM forskolin and 10 μM rolipram (F/R) in the presence 10 μM MG132. Cell extracts were then prepared and immunoblotted with either the PKA substrate phospho-protein, CREB (pSer133), anti-SOCS3 and anti-GAPDH antibodies, respectively. Densitometry of SOCS3 expression from 3 separate experiments is shown in a histogram in the *lower panel*. Significant increases in SOCS3 expression relative to control cells are indicated, ***, p < 0.001, as are non-significant changes (ns).

Having demonstrated that I942 exerts agonist activity in HEK293T-EPAC1 cells we wished to explore whether I942 can regulate down-stream signalling from EPAC1 and Rap1 in HUVECs. We have tried to measure Rap1 and EPAC1 activation in HUVECs using standard assays, but the relative expression levels of both proteins in these cells are so low that it is not technically feasible to detect activation, even with very potent stimuli. However, we have previously shown that activation of EPAC1 in HUVECs, which express EPAC1 but not EPAC2 [52], leads to the induction of the SOCS3 gene through the activation of Rap1, which induces C/EBP transcription factor binding to a key AP1 consensus site in the SOCS3 minimal promoter [48,49,53,54]. We therefore stimulated HUVECs with I942, the Gs-coupled receptor peptide agonist, adrenomedullin 2 (ADM2) [55], rolipram, to inhibit endogenous type 4 cAMP phosphodiesterase activity, or forskolin, to promote adenylate cyclase activation, in the presence of the proteasome inhibitor MG132 to prevent the normally rapid breakdown of SOCS3 protein in cells following its synthesis [56,57] (Fig. 2a). We found that I942 treatment of HUVECs promoted a significant increase in SOCS3 induction and potentiated SOCS3 induction by ADM2 and rolipram, but not forskolin (Fig. 2a). This indicates that I942 activates the same cyclic AMP signalling pathways induced by activation endogenous cyclic AMP signalling systems, as induced by ADM2 and rolipram. Presumably, the high levels of cyclic AMP induced by forskolin treatment induce maximal activation of EPAC1 and SOCS3 induction, and are therefore not stimulated further by co-stimulation with I942. This is interesting because I942 was originally characterised as being a partial agonist, in that although I942 stimulates EPAC1 GEF activity using in vitro assays, it antagonised cyclic AMP-stimulated EPAC1 activity [38]. However, it should be pointed out that the in vitro concentrations of I942 and cyclic AMP used in these competition assays was very high (500 μM), whereas 100 μM I942, as used in the HUVEC experiments here, did promote a significant action of EPAC1 in vitro but not produce a significant inhibition of cyclic AMP-promoted GEF activity [38]. Therefore, in the cell experiments performed here we are able to observe solely the agonist properties of I942 on SOCS3 induction.

To confirm that the induction of SOCS3 by I942 is reliant on EPAC1 activity, we used two methods to inhibit endogenous EPAC1, namely siRNA silencing and use of an EPAC1 antagonist, ESI-09 (Fig. 2b and c). We found that both EPAC1 siRNA and ESI-09 treatment significantly inhibited SOCS3 induction by I942 (Fig. 2b and c), indicating that actions of I942 are dependent on EPAC1. Interestingly, we observed that while ESI-09 treatment completely inhibited I942-induced SOCS3, it did not completely inhibit SOCS3 induction by forskolin and rolipram (F/R), suggesting that the high levels of SOCS3 induction by F/R might also have a PKA component. Indeed, we found that treatment of F/R stimulated cells with the broad-spectrum PKA inhibitor, H-89 [58], to discriminate between EPAC1-dependent and independent actions, partially inhibited SOCS3 induction by F/R (Fig. 2c), suggesting that optimal SOCS3 induction may result from synergistic activation of both PKA and EPAC1. To confirm that H-89 inhibits PKA in HUVECs, cell extracts were also immunoblotted with antibodies towards the transcription factor CREB, which is phosphorylated by activated PKA on Serine 133 [59], and we found that H-89 effectively inhibited PKA in these cells (Fig. 2c). Moreover, we also found that the remaining PKA-independent SOCS3 response, following H-89 treatment of F/R-stimulated cells, coincided with that elicited by I942 (Fig. 2c) and therefore can be attributed to EPAC1 activation.

**Fig. 3:**
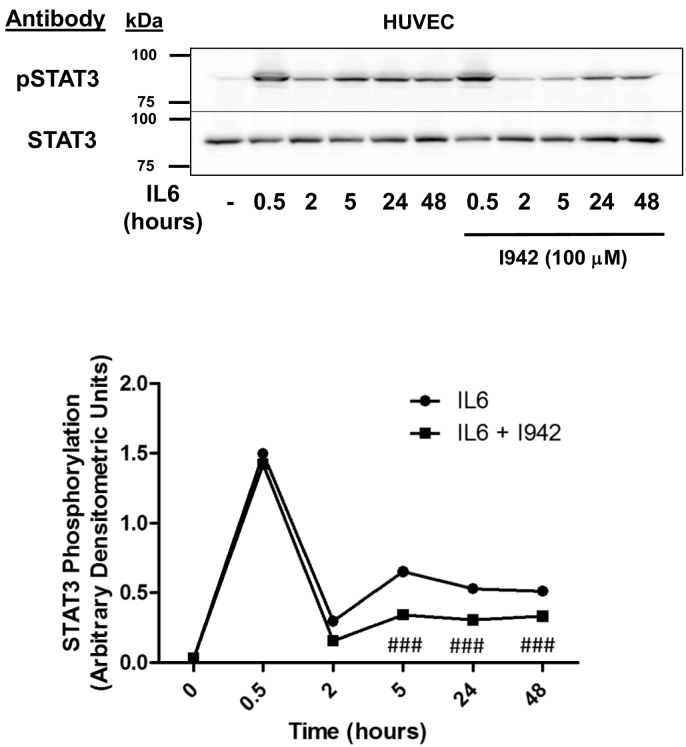
I942 inhibits IL6-promoted JAK/STAT3 signalling in HUVECs. In the *upper panel* HUVECs were pre-incubated with 100 μM I942 for 30 min and then incubated with IL6 (5 ng/ml) plus sIL6Rα (25 ng/ml) for different periods of time up to 48 h. Cell extracts were then prepared and immunoblotted with antibodies to phosphorylated and non-phosphorylated STAT3. Densitometric values from 3 separate immunoblots are shown in the *lower panel* with significant decreases in STAT3 phosphorylation being indicated, ^###^, p < 0.001, relative to IL6-stimulated cells.

Having determined that I942 can induce SOCS3 expression in an EPAC1-dependent manner in HUVECs, we next investigated whether this was able to affect JAK/STAT3 signalling activated by the IL6 receptor (Fig. 3). In these experiments, HUVECs were stimulated with IL6, in the presence or absence of I942, for various times over a time-course of 48 h, following which cell extracts were immunoblotted with anti-phospho-STAT3 antibodies (Fig. 3). We found that co-stimulation with IL6 and I942 caused a significant reduction in STAT3 activation between 5 and 48 h when compared to cells stimulated with IL6 alone for the same period (Fig. 3). Together, these results demonstrate that I942 treatment of HUVECs leads to SOCS3 induction and inhibition of long-term JAK/STAT3 signalling induced by IL6. I942 therefore has the ability to inhibit pro-inflammatory gene expression evoked by JAK/STAT3 signalling in HUVECs.

### 3.3 Identification of genes regulated by I942 in HUVECs

**Fig. 4:**
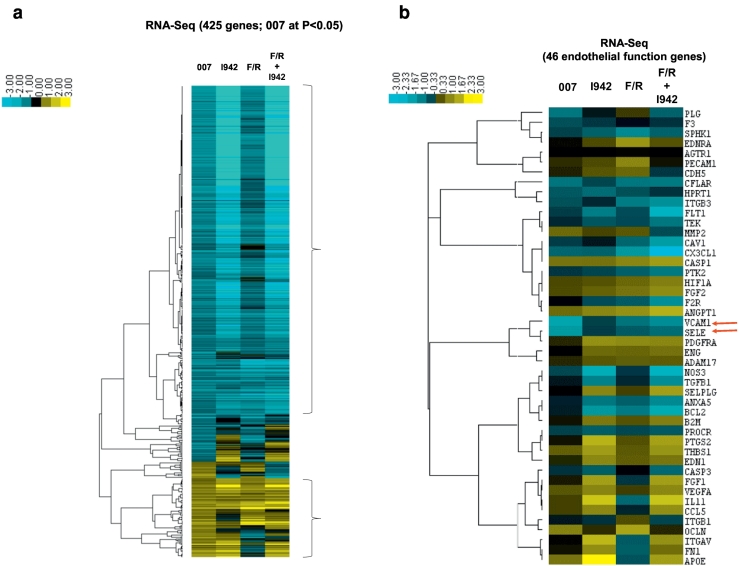

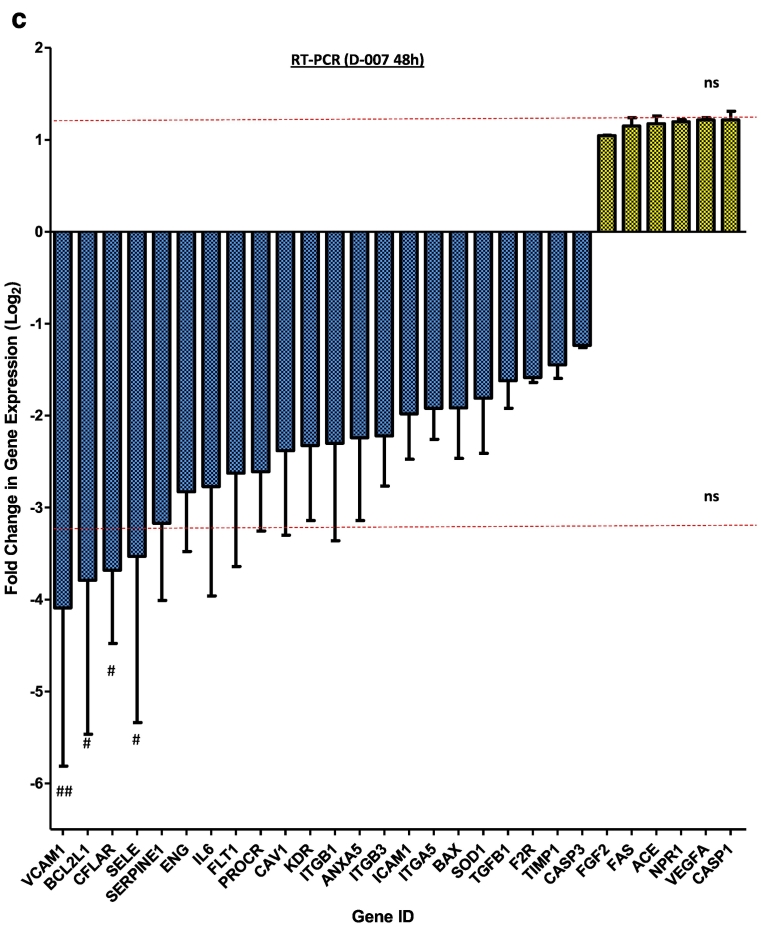
I942 promotes global changes in gene expression in HUVECs. a) In order to identify genes regulated by I942, confluent HUVECs were stimulated for the indicated times with 50 μM 007, 100 μM I942, F/R or F/R plus 100 μM I942. Total RNA was then extracted from cells and processed for RNAseq as described in Materials and Methods. The resulting data was subjected to CLUSTER analysis to generate a dendrogram (on the *left*) to group together gene changes with similar expression profiles. This identified a group of 425 genes that are either significantly (p < 0.05; n = 4) induced (*yellow*) or repressed (*blue*) following treatment. The colour key indicates the relative fold change in gene expression. b) The RNAseq data generated for Fig. 5a was re-analysed to identify gene expression changes that are specifically associated with endothelial function. Red arrows indicated the expression changes for VCAM1 and SELE cell adhesion genes that were strongly suppressed by 007 treatment. c) HUVECs were stimulated for 48 h with 50 μM 007 and then total cell RNA was extracted and subjected to RT-PCR using a RT^2^ Profiler™ PCR Array for Human Endothelial Cell Biology as described in Materials and Methods. The histogram shows fold changes in gene expression of a range of selected genes that were either up-regulated (*yellow*) or down-regulated (*blue*). Significant changes in gene expression are indicated; ^#^, p < 0.05 and ^##^, p < 0.01 (n = 4). The dotted red line indicates gene expression changes that did not achieve significance.

Results suggest that EPAC1 activation by I942 has the potential to suppress the pro-inflammatory gene expression through the inhibition of JAK/STAT3 signalling in HUVECs. However, the full range of genes regulated by EPAC1 has yet to be determined in VECs. To explore this further we aimed to identify EPAC1-regulated genes in HUVECs and determine their responsiveness to I942 treatment. We therefore performed RNA-sequencing (RNA-Seq) in HUVECs treated with 007, I942, F/R or a combination of F/R and I942 for 48 h (Supplementary Data File). From these reads, we identified 425 genes whose activity was significantly (p < 0.05) altered following 48 h 007 treatment and similarly regulated by I942 and F/R, the majority of which were downregulated by the treatments applied (Fig. 4a, blue cluster, and Supplementary Data File). We also found that many of the genes that were regulated similarly by 007, I942 and F/R were specifically involved in vascular function, including the genes for the cell adhesion molecules, VCAM1 and SELE, which were both downregulated and are involved in monocyte adhesion in VECs [11,12] (Fig. 4b; red arrows). To confirm these results we used Human Endothelial Cell Biology RT2 Profiler™ PCR Arrays to examine the expression of endothelial specific genes in HUVEC cells following 007 treatment. The PCR probes included on the array represented candidate genes involved in functions such as inflammation, cell adhesion, platelet activation, angiogenesis, coagulation and apoptosis (Fig. 4c). As with RNA-Seq experiments we found that treatment of HUVECs with 007 for 48 h led to a general suppression of gene expression, although the majority of changes did not reach statistical significance (Fig. 4c). However, we did find that 007 provoked a significant decrease in the expression of VCAM1 and SELE, which we had previously identified by RNA-Seq as being amongst the genes exhibiting the largest fold suppression (Fig. 4b).

### 3.4 I942 suppresses VCAM1 protein expression in HUVECs

**Fig. 5:**
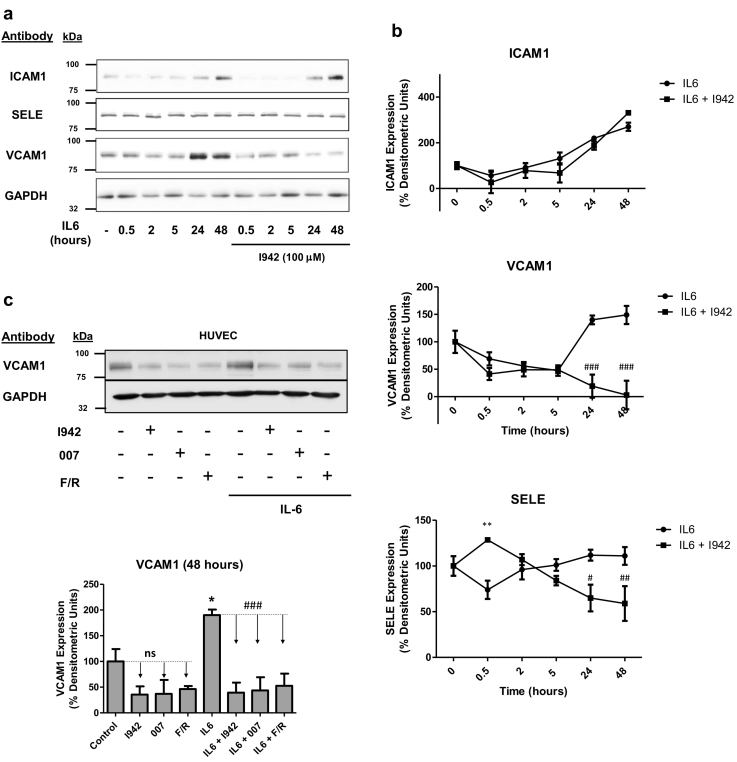
Effects of I942 on the expression of ICAM1, VCAM1 and SELE in HUVECs. a) HUVECs were stimulated for the indicated times with IL6 (5 ng/ml) plus sIL6Rα (25 ng/ml), in the presence or absence of 100 μM I942. Cell extracts were then prepared and immunoblotted with antibodies that recognise ICAM1, VCAM1, SELE and GAPDH. b) Densitometric values from three independent experiments from Fig. 3a are shown as a chart of means ± S.E.M. (n = 3). Significant increases or decreases in protein expression, relative to cells stimulated with IL6 alone are indicated (*, p < 0.05 and **, p < 0.01 or ^#^, p < 0.05 ^##^, p < 0.01 and ^###^, <0.001 respectively). c) HUVECs were stimulated for 48 h with either 100 μM I942, 50 μM 007 or 10 μM forskolin plus 10 μM rolipram (F/R) in the presence or absence of IL6 (5 ng/ml) plus sIL6Rα (25 ng/ml). Cell extracts were then immunoblotted with antibodies to VCAM1 or GAPDH, as indicated. Densitometric values from 3 separate immunoblots are shown in the *lower panel* with significant increases in VCAM1 expression in cells stimulated with IL6 being indicated; *, p < 0.05. Significant decreases in VCAM1 expression relative to cells stimulated with IL6 alone are also indicated ^###^, p < 0.001. Non-significant changes are also indicated (ns).

**Fig. 6:**
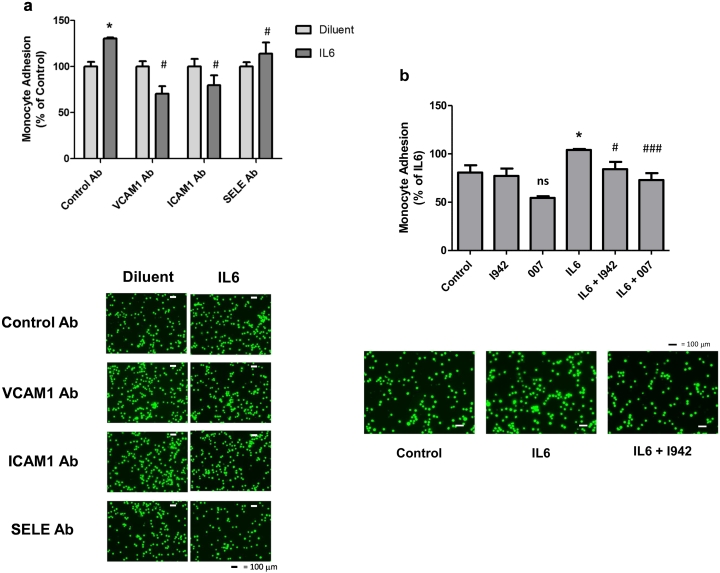

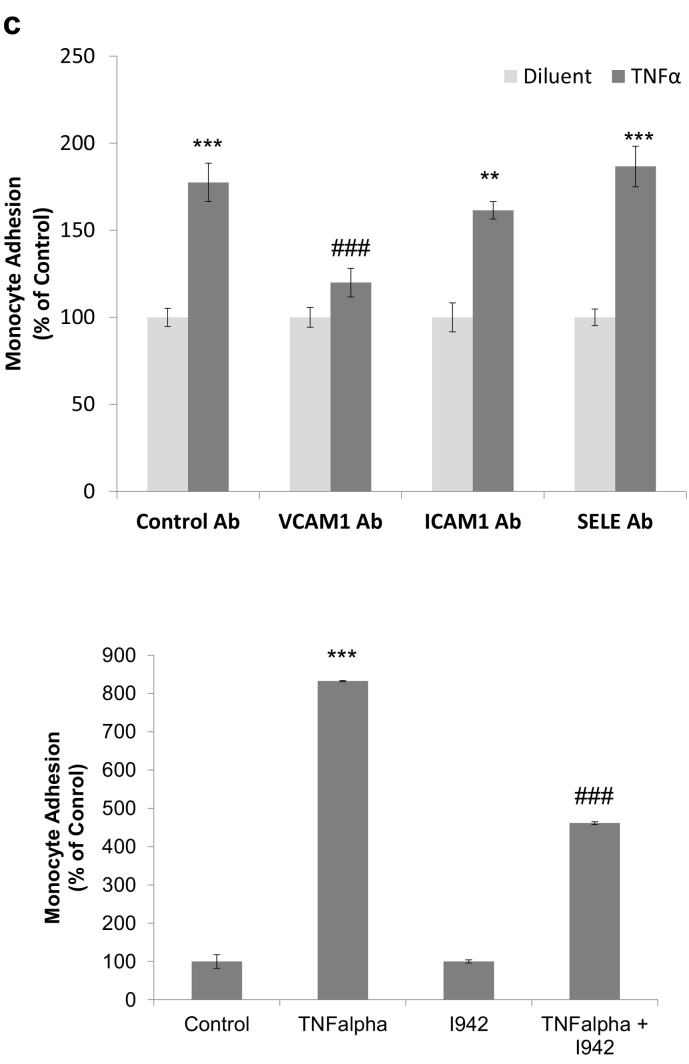
I942 inhibits monocyte adhesion to HUVEC monolayers. a) Confluent HUVEC monolayers were stimulated for 48 h with IL6 (5 ng/ml) plus sIL6Rα (25 ng/ml) in the presence or absence of blocking antibodies to ICAM1, VCAM1 and SELE. Monolayers were then incubated with fluorescently labelled monocytes and the amount of monocyte attachment to monolayers was calculated using a fluorometer, as described in Materials and methods. The relative degree of monocyte adhesion is with significant increases in adhesion being indicated, *, p < 0.05, and significant decreases in adhesion relative to IL6 treated cells with control antibody are also indicated ^#^, p < 0.05 (n = 3). Images of adherent monocytes are shown in the *lower panel*. b) HUVECs were stimulated with IL6 (5 ng/ml) plus sIL6Rα (25 ng/ml) in the presence or absence of 100 μM I942 or 50 μM 007. Increases in monocyte adhesion are indicated, *, p < 0.05, as are significant decreases relative to IL6-stimulated cells, ^#^, p < 0.05 and ^###^, p < 0.001 (n = 3). Non-significant changes are also indicated (ns). Images of adherent monocytes are shown in the *lower panel*. c) In the *upper panel*, confluent HUVEC monolayers were stimulated for 48 h with TNFα (10 ng/ml) in the presence or absence of blocking antibodies to ICAM1, VCAM1 and SELE. Monolayers were then incubated with fluorescently labelled monocytes and the amount of monocyte attachment to monolayers was calculated using a fluorometer, as described in Materials and methods. The relative degree of monocyte adhesion is with significant increases in adhesion being indicated, **, p < 0.01 and ***, p < 0.001, and significant decreases in adhesion relative to TNFα treated cells with control antibody are also indicated ^###^, p < 0.001 (n = 3). In *the lower panel*, HUVECs were TNFα (10 ng/ml) for 5 h in the presence or absence of 100 μM I942. Increases in monocyte adhesion are indicated, ^###^, p < 0.001 (n = 3), as are significant decreases relative to TNFα -stimulated cells, ^###^, p < 0.001.

Given that the expression of VCAM1 and SELE genes is significantly downregulated following EPAC1 activation by 007, I942 and F/R in HUVECs (Fig. 4b), we next examined whether I942 could exert similar effects on VCAM1 and SELE protein levels. We also examined the expression of ICAM1 protein, since changes in its expression is often linked to inflammatory responses. We therefore treated HUVECs with IL6 over a time-course of stimulation up to 48 h, in the presence or absence of I942. Cell extracts were then prepared and immunoblotted with antibodies recognising the adhesion molecules ICAM1, VCAM1 and SELE (Fig. 5a). We found that IL6 treatment led to a robust induction of ICAM1 and VCAM1 protein expression at 48 h, but exerted little effect on SELE expression (Fig. 5a and b). Co-incubation with IL6 plus I942 led to a significant reduction in the expression of VCAM1 and SELE, but not ICAM1, in IL6-treated cells (Fig. 5b). We confirmed an involvement of EPAC1 activation in the suppression of VCAM1 protein levels by stimulating cells with I942, 007 and F/R for 48 h, in the presence or absence of IL6 (Fig. 5c). This led to a general suppression of VCAM1 protein expression both in the presence and absence of IL6, indicating a negative regulatory role for EPAC1 in the basal control of VCAM1 expression in HUVECs. To determine whether the regulation of expression of VCAM1 or SELE by I942 and 007 can influence the attachment of monocytes to HUVECs, we stimulated cells for 48 h with IL6 in the presence or absence of I942 or 007 and determined the ability of THP-1 monocytes to attach to HUVEC monolayers (Fig. 5d). Monoclonal blocking antibodies against ICAM1, VCAM1 and SELE were also used to determine their individual roles in promoting monocyte adhesion (Fig. 6a). Results demonstrated that IL6 promoted a significant recruitment of monocytes to HUVEC monolayers. This was blocked by co-incubation with ICAM1, VCAM1 and SELE antibodies (Fig. 6a). We also found that co-incubation with either I942 or 007 suppressed IL-6 driven monocyte adhesion (Fig. 6b). Given that I942 and 007 suppresses VCAM1 protein expression (Fig. 5c), this suggests that EPAC1 regulates monocyte adhesion through downregulation of the VCAM1 gene in VECs.

Although these experiments indicate that both I942 and 007 can inhibit IL6-promoted monocyte adhesion to HUVECs, which correlates with inhibition of VCAM1 expression by both these agents (Fig. 5c), it is unclear as to whether suppression of VCAM1 expression is sufficient to block monocyte adhesion in these cells. To test this we examined the role of VCAM1 in TNFα-mediated monocyte adhesion and found that inhibition of VCAM1 with blocking antibodies is sufficient to inhibit TNFα-mediated monocyte adhesion in HUVECs (Fig. 6c). Moreover, we found that I942 treatment significantly inhibited TNFα-mediated monocyte adhesion, presumably through inhibition of VCAM1 expression.

## 4 Discussion

Our aim has been to develop potent, small molecule selective regulators of EPAC1 activity to determine the role of EPAC1 in the control of vascular inflammation and cardiovascular disease by cyclic AMP. To date, high throughput screening (HTS) of compound libraries to search for small molecule regulators of EPAC activity [[60], [61], [62], [63]] has led to the discovery of the EPAC2-selective inhibitor, ESI-05 [64] and a non-selective EPAC1 and EPAC2 inhibitor, ESI-09 [[65], [66], [67]]. Subsequently, uncompetitive (CE3F4) and non-competitive EPAC1 inhibitors (5225554 and 5376753) have also been identified through HTS using an in vitro EPAC1 GEF activity assay [61] and an EPAC-based bioluminescence resonance energy transfer-based assay [60], respectively. Notably, only two selective cyclic nucleotide derived EPAC agonists have been developed, namely the EPAC1 selective agonist, 007 [68], and the EPAC2 agonist, S-220 [68]. Unfortunately, the practical use of S-220 in vivo is limited, since activation of EPAC2, but not EPAC1, causes arrhythmia and reduced cardiac function in animal models [69,70] and moreover, 007 has limited cell permeability and potency in vivo. There is therefore a pressing need to develop further non-cyclic nucleotide EPAC1-directed agonists in order to fully define the role of EPAC1 in the development of CVD.

To address this, we previously carried out HTS of a 5000 compound, small molecule library to isolate ligands targeting the CNBD of EPAC1 [38]. After triage, using microscale thermophoresis (MST), we identified a range of small molecule competitors of cAMP binding to EPAC1, with potencies similar to that of cAMP (pIC50 = 5.5). Subsequently, ligand observed nuclear magnetic resonance (NMR) analysis demonstrated direct interaction between inhibitor I942 and the CNBD of EPAC1 [38]. We also found that I942 effectively promotes EPAC1, but not EPAC2, activity in vitro [38], but we had yet to determine the cellular actions of this compound. Here, for the first time, we demonstrate that I942 has the ability to provoke EPAC1 activation in HEK293T cells expressing EPAC1, using activation-specific EPAC1 antibodies and Rap1 activation assays (Fig. 1). I942 therefore represents an effective tool to probe the function of cellular EPAC1. In this regard, and consistent with our previous observations that activation of EPAC1 is sufficient to induce SOCS3 gene activity [49,53,54], we found that I942 promotes SOCS3 protein induction in an EPAC1-dependent manner (Fig. 2b and c) as well as inhibiting IL6-promoted STAT3 activity. Activation of EPAC1 by I942 therefore has the potential ability to regulate gene expression both in the short-term and long-term. In the case of the SOCS3 gene, there are direct effects on transcription factor recruitment to the gene promoter between 30 min and 5 h [49,54], whereas, in the long-term, I942 inhibits STAT3 signalling between 5 and 48 h (Fig. 3). One caveat does remain however, in that we have tried to measure Rap1 and EPAC1 activation in HUVECs using standard assays, but the relative expression levels of both proteins in these cells are so low that it is not technically feasible to detect activation, even with very potent stimuli. Future work should therefore be directed to determining any off-target effects of I942 in these cells, since, at this stage, we cannot equivalently state that induction of SOCS3 and suppression of STAT3 activation are wholly due to I942 effects in these cells.

In the present study, we have found that treatment of HUVECs with I942 leads to alterations in the expression of a wide variety of genes, many of which are associated with vascular function. In particular, we demonstrate that both 007 and I942 suppress the expression of the pro-inflammatory adhesion molecule, VCAM1, at the protein and mRNA level. This is consistent with previous work demonstrating that the PDE4 inhibitor, roflumilast, inhibits neointimal hyperplasia and VCAM1 expression in an EPAC1 dependent manner in vascular smooth muscle cells [71] and that the cyclic AMP-elevating drugs, forskolin and IBMX, suppress the expression of VCAM1 in HUVECs [72], although the molecular mechanisms underlying these effects remain to be determined. However, given that the IL6-activated JAK/STAT3 pathway leads to the upregulation of VCAM1 expression in human aortic endothelial cells (HAECs) [18], it is tempting to speculate that inhibition of JAK/STAT3 signalling by 007 [56] or I942, as reported here (Fig. 3), may play a role in regulating VCAM1 expression. However, regulation of other transcription pathways in addition to JAK/STAT3 signalling may underlie the actions of I942 on the expression of cell adhesion molecules. In this regard, the VCAM1 gene promoter contains binding sites for a number of transcription factors, including NF-κB and AP-1, both of which can be activated by IL6 [[73], [74], [75]]. AP-1 transcription binding sites can bind C/EBP and c-Jun transcription factors, both of which we have shown to be important for SOCS3 gene induction by EPAC1 [49,53,54]. Equally, it has been known for some time that cyclic AMP inhibits NF-κB activation in a variety of cell backgrounds, including endothelial cells [[76], [77], [78], [79]], but the role of EPAC1 in the regulation of NF-κB remains to be determined since it has been reported to serve as an activator of NF-κB signalling [80,81]. Clearly, further work will be necessary to determine the molecular mechanisms underlying the actions of I942 on adhesion molecule expression in VECs. However, it is nevertheless clear that I942 represents a valuable tool for probing the actions of EPAC1 in vascular function. I942 therefore represents the first non-cyclic nucleotide EPAC1 agonist with the potential to exert anti-inflammatory actions in VECs. Indeed, this work contributes to the case justifying an exploration of EPAC1 activators, based on the starting structure of I942, as agents for suppression of vascular inflammation and neo-intimal hyperplasia and promotion of re-endothelialisation following percutaneous coronary intervention procedures, where EPAC1 appears to play a key role.

## Declarations of interest

There are no conflicts of interest.

## Funding information

This work was funded by a British Heart Foundation (BHF) project grant (BHF grant number PG/15/15/31316) awarded to SJY and GH. BvB is a James Watt Research Scholar and ULS is funded by a BHF scholarship (BHF studentship number FS/17/12/32703).

## Transparency document

Transparency document Image 1
